# The Surgical Club of South West England

**Published:** 1983

**Authors:** 


### The Surgical Club of South West England

Abstracts of Papers given at the Autumn Meeting 1982 at Bath, October 22–23

### INTERVENTIONAL RADIOLOGY IN A DISTRICT GENERAL HOSPITAL

**A. H. Chalmers, Bath**

Interventional radiology refers to the more invasive techniques being applied in diagnostic radiology as well as the therapeutic procedures which are performed by radiologists.

In the vascular system embolisation is used preoperatively to decrease tumour vascularity or can be used palliatively in patients with functioning metastases or painful hepatomegaly. Gastro-intestinal bleeding or bleeding from elsewhere after surgery or trauma can be localised and controlled using a variety of embolic agents or drugs.

Percutaneous arterial angioplasty had increased enormously in the last 4 years and has a high success rate for iliac stenoses as well as stenoses in femoral, renal and coronary arteries. For the claudicating patient angioplasty offers effective treatment at a time when surgery is not yet indicated.

In the biliary tract percutaneous catheterisation allows internal or external drainage of an obstructed biliary system. A catheter across a bile duct stricture can be replaced with an endoprosthesis. Percutaneous transhepatic gallstone removal has been performed but of wider application is the removal of gallstones from the biliary tree via a T-tube track after surgery. A 14 French T-tube preferably inserted fairly laterally gives the radiologist the best approach.

Drainage of obstructed renal tracts can be performed using similar techniques and the percutaneous removal of renal pelvic stones is now being undertaken jointly by radiologists and surgeons.

Interventional radiology is an exciting new branch of radiology but requires close co-operation between radiologists, physician and surgeon to realise its full potential.

### ULTRASOUND GUIDED FINE NEEDLE ASPIRATION BIOPSY

**M. J. Noakes, Bath**

Percutaneous fine needle aspiration biopsy can be undertaken using the imaging modalities of fluoroscopy, C. T. scanning and ultrasound for guidance. Ultrasound guided techniques were described and illustrated with examples of intra-abdominal lesions which were or could have been biopsied.

Real-time and static B scan apparatus may be used although the use of real-time is becoming more popular. Specially adapted transducers are available but are not essential.

Having localised the lesion a 22- or 23-gauge needle is introduced percutaneously under aseptic conditions with prior local anaesthesia and the progress of the needle is observed when possible on the ultrasound image. Biopsy transducers make this observation easier. In obese patients a larger needle through which the fine needle can be passed may be used to stabilise the fine needle in the superficial tissues.

With the tip of the needle in the lesion a sample is obtained by aspiration using a 20 ml syringe, sampling from the whole depth of the lesion. The aspirated material is expelled onto a microscope slide and a smear is made for expert cytological interpretation. Such expertise is fundamental to the procedure.

No serious complication has been reported from the use of the technique.

### PARATHYROID VENOUS SAMPLING

**D. A. B. Dunlop, Bath**

Hyperparathyroidism is a serious and potentially crippling disease; *but*, it is undoubtedly curable by surgery. However, the causative adenoma may be small and difficult to locate in the bloody battleground of the neck. Even those surgeons who claim excellent results without pre-operative localisation of the tumour admit that the operation is arduous and time-consuming . . .. In less skilled hands, the quest is fruitless and the operative complications alarming.

Methods of pre-operative localisation such as plain film, barium swallow, isotope studies, thermography, ultrasound and computed tomographic scanning are insufficiently sensitive to locate small adenomas. Thyro-cervical arteriography is not without risk to the spinal cord.

For the past decade sampling of the veins draining the parathyroid glands for assay of parathyroid hormone content has been recognised as *the* most successful means of pre-operative localisation. Sampling the maximum number of small veins, in addition to the large neck veins, greatly increases the accuracy of tumour localisation. Thus, up to 30 blood samples are taken for hormone assay and simultaneous venography is performed to localise and record the sampling site. Venography facilitates the identification of small vessels and reveals idiosyncrasies of venous flow specific to each patient.

The parathyroid glands usually drain into the thyroid venous plexus which itself drains to the internal jugular veins via the superior and middle thyroid veins, and to the innominate veins via the inferior thyroid veins. Each gland tends to drain ipsilaterally and inferiorly. Variations with contralateral flow both anteriorly via the anterior jugulars, and posteriorly via the vertebral plexus frequently occur even in patients who have not undergone neck surgery.

*Technique:* A 6 French gauge Kifa catheter with a simple curve and small side hole is passed by Seldinger technique via the right femoral vein, IVC and SVC to the right internal jugular. The right superior thyroid vein is then catheterised, sampled and a contrast injection made. This venogram details much of the thyroid plexus architecture and demonstrates the site of the middle and inferior thyroid vessels. Samples are taken from the right internal jugular at different levels. The procedure is repeated on the left side. The inferior drainage is next catheterised and sampled. This is much more difficult but important because lower pole tumours are four times more common and even upper pole tumours tend to drain maximally via the inferior veins.

The inferior thyroid venous drainage is variable. In 60% of the population the two inferior thyroid veins unite to form a single common trunk which usually enters the low left innominate vein. At least 10% of the population display idiosyncratic inferior thyroid venous drainage; when the hormone levels are subsequently evaluated these apparently trivial variations in the standard anatomy can be of great significance.

Adenomas are situated in the mediastinum in about 5% of cases; therefore, the azygos, hemiazygos and thymic veins are also sampled.

Occasionally (4%) venograms will actually demonstrate an adenoma.

PVS offers successful pre-operative localisation in up to 88% of cases.

The mid line radiation dose is between 7 and 21 cGy, depending upon the screening time.

---

### Short Papers for the South West Surgical Prize

### OESOPHAGEAL CARCINOMA IN BATH

**D. CRANSTON Bath**
*Sponsor:* **D. C. Britton**

The management of carcinoma of the oesophagus remains a difficult problem, where the best treatment is debatable, the operative mortality high, and the prognosis in the Western hemisphere uniformly bad.

Fifty-two patients have had oesophagogastrectomies in Bath in the 10-year period between 1968 and 1978. Twenty-six (50%) had oesophagogastrectomies for adeno carcinoma, 26 for squamous cell carcinoma. Post-operative mortality was 42% with a 35% 1-year survival, and 15% 2-year survival. Eleven of the twenty-two post operative deaths occurred from anastomatic leakage.

Seventy-four patients had Celestin intubation for carcinoma between 1976 and 1981; 36 inserted at laparotomy, 38 by endoscopy. All these patients were rejected from major surgery, being too ill, too old, or with metastatic disease. There was a 5% mortality from endoscopic intubation as compared with a 31% mortality from laparotomy. Ten per cent survived 1 year, and 2 patients were alive at 2 years.

The quality of life with a Celestin tube in situ was assessed. Ninety per cent were able to eat semi-solid foods. Seventy-five per cent had infrequent blockage of the tube. Eighty-five per cent of the patients felt the Celestin tube improved their quality of life.

The place of radiotherapy has yet to be fully assessed but it may well have an important role to play in the treatment of squamous cell carcinoma of the oesophagus.

(Judged to be the winning paper.)

### THE 5-YEAR RESULTS OF HIGHLY SELECTIVE VAGOTOMY

**R. H. Kennedy, Bristol**

The 5-year results of highly selective vagotomy (HSV), performed at the Bristol Royal Infirmary, have been reviewed to assess the ulcer recurrence rate and side effects.

Sixty patients (M 44, F 16, mean age 50 years) who underwent HSV were followed prospectively for 5–8.7 years. Operation was for duodenal ulcer (DU) in 47 cases and gastric ulcer (GU) in 13 cases.

Ulcer recurrence in the DU group was 8.5%. However, the recurrence rate following an adequate lower oesophageal denervation, of at least 5 cm ($n$=43), was 4.7%. The incidence of side effects in the DU group was the same as that reported by Goligher (1978), there being no cases of troublesome post-vagotomy diarrhoea. Seventy-seven per cent had an excellent or good result (21 Visick I, 15 Visick II). In the remaining DU patients (4 Visick III, 7 Visick IV), there were 4 recurrent ulcers and 2 patients requiring further surgery; for lesser curve necrosis and gastric stasis.

HSV was performed for gastric ulceration with excision of the ulcer ($n$=7) or biospy ($n$=4). One recurrence occurred and 92% were Visick grade I or II.

HSV is, therefore, a safe and effective operation for peptic ulcer disease accompanied by a small incidence of side effects.

### THE USE OF INTRAVESICAL DIMETHYL SULFOXIDE (DMSO) IN THE TREATMENT OF INTERSTITAL CYSTITIS—PRELIMINARY REPORT

**N. Anandan, (*Sponsor:* J. C. Gingell), Bristol**

Interstitial cystitis remains a therapeutic challenge to the Urologist. It is a chronic distressing condition affecting almost exclusively women. The cause is unknown, and a variety of treatment regimes have been employed to relieve severe urgency, frequency and bladder pain. Currently, the most popular method of treatment is that of intermittent forceful distension of the bladder under anaesthesia, together with steroid therapy. This does not always prevent some patients requiring bladder denervation, bladder transection and enhancement with colon or caecum and occasionally urinary diversion.

Several recent favourable reports from America on the use of intravesical DMSO in a variety of inflammatory disorders of bladder, including interstitial cystitis, prompted us to look at the use of this agent in our patients.

After an initial detailed urological history, the diagnosis was established by cystoscopy, endoscopic photography, mucosal biopsies and measurement of bladder volume. Fifty ml of 50% DMSO was instilled into the bladder by catheter every 2 weeks, and the symptoms were recorded at each visit. Ten patients have now completed 6 months' treatment, and 8 of these have undergone. endoscopic reassessment.

There was symptomatic improvement in the majority of patients; good (3 cases), fair (6 cases), no change (1 case). Analysis of frequency/volume charts, completed by the patients, showed improvement in both daytime and night frequency in 2 cases, improvement in day frequency in 2 cases, improvement in nocturnal frequency in 2 cases, and no improvement in 4 cases. Endoscopic reassessment showed mild improvement in the bladder mucosal appearance in 1 case, marked improvement in 1 case, and the appearances were unchanged in 6 cases. The bladder capacity was increased by 150 ml in 3 cases, by 50 ml in 3 cases, and unchanged in 3 cases.

The only significant side effect was that of a delayed hypersensitivity skin eruption in 1 patient after 9 treatments. This has not previously been reported. All patients had an odour of garlic on their breath. Intravesical DMSO is a useful palliative outpatient treatment for the symptomatic relief of patients with interstitial cystitis.

### EXPERIENCES OF THE FIRST YEAR OF AN OPEN ACCESS DYSPHAGIA CLINIC

**D. C. Britton, K. R. Gough and E. Wilkins, Bath**

Dysphagia is an unpleasant symptom which requires emergency treatment. An open access dysphagia clinic was therefore set up in the Bath Health District. The clinic is held weekly in the endoscopy department. Any patient with dysphagia for solid food for longer than 1 week can be referred. The patients attend fasting. A full history and clinical examination is taken. This is followed by barium swallow, and endoscopy on the same day.

In the first year 90 patients were referred with 'true dysphagia'. Of these, 61 described dysphagia for solids only, and 29 dysphagia for solids and liquids. Definite abnormalities were found in 77 of the 90 patients who described 'true dysphagia'. Fifty-five patients had peptic or malignant strictures. Oesophageal dysmotility was diagnosed in 6 patients. Other diagnosis included a pharyngeal pouch (1), post vagotomy stricture (1), post cricoid web (1). In the remaining 13 patients, no definite abnormality was found.

Nineteen other patients were referred in the first year with an equivocal history of dysphagia. Five were found to have organic abnormalities: hiatus hernia with oesophagitis (2), gastric ulcer (3) and oesophagitis with duodenitis (1).

Twenty-three of the cases with stricture were found to be caused by malignancy. Twelve of these patients had endoscopic per-oral oesophageal intubation immediately, and were discharged within 36 hours of the procedure. Six patients were referred for definitive surgery. The other 5 patients had oesophageal dilatation, and were supplied with a Hurst's bougie for intermittent dilatation at home.

Our experience with an open access dysphagia clinic is encouraging. Significant lesions were found in 25% of the patients. The average period between general practitioner and formal clinic appointment was only 7 days. Our clinic speeds up referral and treatment of an unpleasant emergency symptom, and can be recommended as an efficient and effective service to patients in distress.

### CURRENT INVESTIGATION OF GASTRO-OESOPHAGEAL REFLUX

**R. B. Smith, Bath**

The diagnosis of abnormal reflux from stomach to oesophagus can be difficult. Symptoms alone are an unreliable guide. Conventional radiology has a poor correlation with symptoms and up to a 25% false postive incidence. More objective tests are available but require expensive and sophisticated equipment. The most popular such tests are the standard acid reflux test and 24 hour pH monitoring. Both these tests measure intra-oesophageal pH via a probe placed above the lower oesophageal high pressure zone. The latter must be accurately located by oesophageal manometry. The standard acid reflux test involves provoking the reflux of 0.1 N HCl placed directly into the stomach by coughing, breathing, the valsalva and the Mueller manoeuvres in four different positions. Scores of three or more constitute abnormal reflux. This test correlates well with the severity of symptoms but has a false positive incidence of around 10%. Twenty-four hour pH monitoring combines the virtues of an endogenous acid perfusion test, a test of the type of reflux material and a record of the position in which reflux can occur. It is the only quantitative objective test of reflux. Radioisotopes may well hold the key to non-invasive tests of reflux particularly since the advent of short half life isotopes, large collimators and computerised recording and imaging facilities.

Only by the use of objective methods of assessment can good case selection for antireflux surgery be achieved.

### PARATHYROID IDENTIFICATION

**A. R. Turnbull, Bath**

The parathyroids may be selectively stained by methylene blue. This technique was assessed in a series of 22 parathyroidectomies. The patients' ages ranged between 19 and 77 with a mean age of 58 years, and included 15 females (71%). The classical symptoms of 'Bones, Groans (Abdominal), Moans (Psychic) and Stones' occurred in a similar proportion of patients. In addition, polydypsia, polyuria, lethargy and constipation were noted. The diagnosis was confirmed by a raised Ca, a low $PO_4$ and a raised PTH level. Methylene blue, in a dose of 5–10 mg/kg, was infused in 500 ml N/S in the hour prior to surgical exposure. Adenomata and hyperplastic glands were selectivity stained. The superior glands were invariably found in the normal anatomical position, but the inferior glands were often ectopic, and four were found in the superior mediastinum, with a further two within the thymus. Seventy-five per cent of the patients had one or more adenomata, and there was one carcinoma. Most had a transient asymptomatic drop in the Ca level post-operatively between 24 and 72 hours, but 4 patients suffered a prolonged hypocalcaemia. Vital staining with methylene blue enabled rapid and positive identification of the glands, particularly when they were ectopic, and there were no complications from its use.

### ALPHA BLOCKERS AND THE BLADDER NECK

**C. W. Lott and C. A. C. Charlton, Bath**

Alpha-adreno receptors (found in the trigonal area and prostatic capsule) are the dominant neurological component in opening of the bladder neck. There are a few beta-adreno receptors present and a minimal number of cholinergic parasympathetic fibres have been identified. Stimulation of the alpha-adreno receptors leads to a rise in the prostatic urethal pressure, and pharmacological blockade facilitates the opening of the bladder neck and upper urethra. Such a drug is Phenoxybenzamine, and 10 mg was given nightly to 50 patients who had been put on the waiting list for relief of lower urinary tract obstruction.

The drug is contra-indicated in those with postural hypotension, as judged by a 20 mm of Hg drop in their systolic blood pressure measurements on standing and lying down. Similarly, those with a history of cardio-vascular disorders (including those on antihypertension therapy) were excluded from this trial. One patient failed to comply, and 30 had varying degrees of side effects as judged by symptoms of postural hypotension, stuffiness of the nose and tightness of the chest, and other lesser complaints. In 15 patients the symptoms were severe enough to make them discontinue the drug. In 60% of all the 50 cases treated, patients reduced their frequency by day and night, and the severity of the urgency of micturition was impressive. Whereas, before treatment some 10 patients had suffered from moderate to severe urgency, this number fell to 2 patients; and the number with no urgency increased from 24 to 40 patients. Of these 31 patients who voided less than 200 ml prior to treatment, there occurred a significant increase in their maximum flow rate (mean of 7.61 ml/second to 11.38 ml/second, $P<0.01$) and in the remaining 15 patients (measurements were not done in 4 patients) whose pre-treatment voiding volumes exceeded 200 ml, the flow rate was also significantly increased.

This drug should be considered in patients who develop acute retention following some operations, e.g. hernia, piles and hip operations; those in whom prostatectomy is inadvisable or being postponed for some reason, and finally in those who surprisingly develop retention with no previous symptoms of prostatism.

### DEVELOPMENT OF A MODERN HOSPITAL

W. F. W. Southwood, Bath

In 1976 the Bath Consultants decided to try to improve the facilities for private hospital accommodation in the city. The initial plan was to try to upgrade the existing Nursing Home by raising money from voluntary contributions; this would have entailed closure for at least 12 months which was not considered to be acceptable. Great difficulty would have been encountered in raising the necessary funds and this project was abandoned.

A specialist in private hospital development came to Bath in 1978 and after initial consultatations was invited to carry out a feasibility study for the building of a new hospital. This proved favourable and showed that the Clinical Area could support a new private hospital of 50 beds, each with a private bath or shower and toilet facilities. There was sufficient work to support two major theatres and one minor theatre. An x-ray department, physiotherapy department and a pathological laboratory would also be included.

After considerable difficulty a 5½ acre site was found on the southern side of the city and planning permission was obtained, both from Bath City Council and Avon County Council. The design of the hospital included a high-dependency nursing area adjacent to the theatre complex; all patients having an operation would recover in this area and major operative cases will probably stay there for 2 or 3 days. In addition this area would be used for seriously ill medical patients, and would contain all necessary monitoring equipment and be situated close to the pathology laboratory. A resident medical officer is to be appointed.

